# High-Performance CsPbI_2_Br Perovskite Solar Cells with Zinc and Manganese Doping

**DOI:** 10.1186/s11671-019-2936-8

**Published:** 2019-04-02

**Authors:** Ubaid Khan, Yu Zhinong, Abbas Ahmad Khan, Almas Zulfiqar, Naeem Ullah

**Affiliations:** 1 School of Optics and Photonics, Beijing Engineering Research Center of Mixed Reality and Advanced Display, Beijing Institute of Technology, Beijing, 100081 China; 2 Institute of Chemistry, Chinese Academy of Sciences, Beijing, 100190 China; 3 School of Optics and Photonics, Beijing Institute of Technology, Beijing, 100081 China

**Keywords:** Perovskite solar cell, Defect density, Stability, CsPbI_2_Br, ZnCl_2_-MnCl_2_ doping

## Abstract

Photovoltaic performances of CsPbI_2_Br solar cells are still lower than those of hybrid inorganic–organic perovskite solar cells, and researchers are exploring ways to improve their efficiencies. Due to its higher thermal stability in comparison with the generally studied hybrid inorganic–organic perovskites, all-inorganic CsPbI_2_Br has recently attracted great attention. By utilizing the combination of MnCl_2_ and ZnCl_2_ particles doping to modulate film growth, it is found that MnCl_2_ and ZnCl_2_ particles infiltrate into the holes of the CsPbI_2_Br lattice through the growth procedure, leading to suppressed nucleation and reduced growth rate. The combination assists to achieve higher CsPbI_2_Br crystalline grains for increased *J*_sc_ as high as 15.66 mA cm^−2^ and FF as large as 73.37%. It is indicated that a specific combination of ZnCl_2_-MnCl_2_ doping can fundamentally improve the film surface morphology, reduce trap density, and suppress the recombination of carriers. Consequently, power conversion efficiency (PCE) is significantly improved from 13.47 to 14.15% compared with the reference device without doping.

## Introduction

Hybrid organic–inorganic perovskites have aroused great concerns because of their excellent electronic and optical properties [1–7] such as high mobility of the charge carriers and tunable band gap [8–11]. Notably, the power conversion efficiency (PCE) of perovskite-based organic–inorganic hybrid solar cells has improved from 3.8 to 23.3% through the cation exchange [12–17]. There are still challenges to overcome all environmental degradation [18]. Up to now, the cesium lead halide perovskite solar cells have been researched by many groups [19–22]. The large band gap of CsPbBr_3_ is about 2.3 eV, which is too large to absorb long-wavelength lights [23, 24]. The CsPbI_3_ has a low band gap of 1.73 eV, but it degrades rapidly from black phase to yellow phase at ambient temperature [25, 26]. CsPbI_2_Br perovskite shows a desirable band gap of 1.91 eV and is stable in the black phase in ambient air [19, 20]. It is demonstrated that the size of microcrystalline grain is a key factor for increasing the efficiency of solar cell. [27–30]. It appears that the grain boundaries in the surface of perovskite film suppress the recombination of the charges in their trap states [31]. Meanwhile, grain boundaries can provoke external states near the edge of the valence band which will impede the spread of the hole [32]. Therefore, it is desirable that the CsPbI_2_Br has a huge particle size and a low trap charge density [33]. For this purpose, the doping of impurities was explored extensively by incorporating several ions into the host lattice to modulate the performance of the film [34]. For example, by incorporating potassium into CsPbI_2_Br, these large CsPbI_2_Br crystallites could be obtained to improve the formation of charge carriers and the better charge transport increases PCE [35]. Chu et al. used KCl as an additive material to obtain uniform and dense MAPbI_3_ perovskite films with large grain-size nanocrystals [22]. Liu et al. reported that the addition of Mn^2+^ with a certain amount could significantly improve the crystalline grain size and achieve superior solar cell performance [36]. All-inorganic CsPbI_2_Br has recently attracted great attention due to its higher thermal stability in comparison with the generally studied hybrid inorganic–organic perovskites. In the paper, it is indicated that a specific combination of ZnCl_2_-MnCl_2_ doping can fundamentally improve the film surface morphology, reduce trap density, and suppress the recombination of carriers. Consequently, PCE is significantly improved from 13.47 to 14.15% compared with the reference device without doping. To the best of our knowledge, the PCE of 14.15% is among the best performance of CsPbI_2_Br perovskite solar cells.

## Results and Discussion

We prepared 1.0 M using solution CsBr together with equal stoichiometric PbI_2_ in mixed solvents of DMF and DMSO as the precursor solution. Through a one-step spin-coating method, a 350-nm film (measured by profilometer) was obtained after being annealed at 150 °C. To study the effect of additive on the film morphology and the device performance, we incorporated different contents of ZnCl_2_-MnCl_2_ (0%, 0.25%, and 0.50%) molar ratio, marked by CsPbI_2_Br-0%, CsPbI_2_Br-0.25%, and CsPbI_2_Br-0.50%, respectively, into the CsPbI_2_Br precursor solution.

**Fig. 1:**
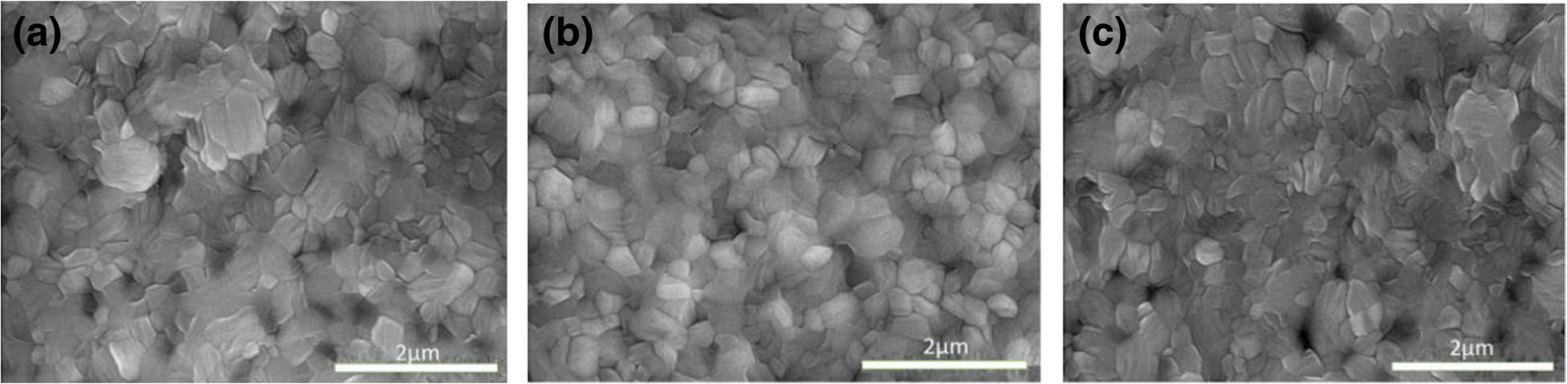
The top-view SEM images of the CsPbI_2_Br-ZnCl_2_-Mncl_2_ films. **a** CsPbI_2_Br-0%. **b** CsPbI_2_Br-0.25%. **c** CsPbI_2_Br-0.50%

Figure 1a–c shows the top-view of CsPbI_2_Br films with different levels of ZnCl_2_-MnCl_2_. It can be seen that when the combination of ZnCl_2_-MnCl_2_ content is less than 0.25%, the CsPbI_2_Br film becomes more uniform and compact with the increase of the ZnCl_2_-MnCl_2_ content. In addition, there are almost no pin holes in the CsPbI_2_Br-0.25% film, suggesting that the combination of ZnCl_2_-MnCl_2_ dopants is in favor of surface morphology of films. In the CsPbI_2_Br-0.50%, however, small pin holes emerge in the film, which can create derivation paths and result in worse device performance.

**Fig. 2:**
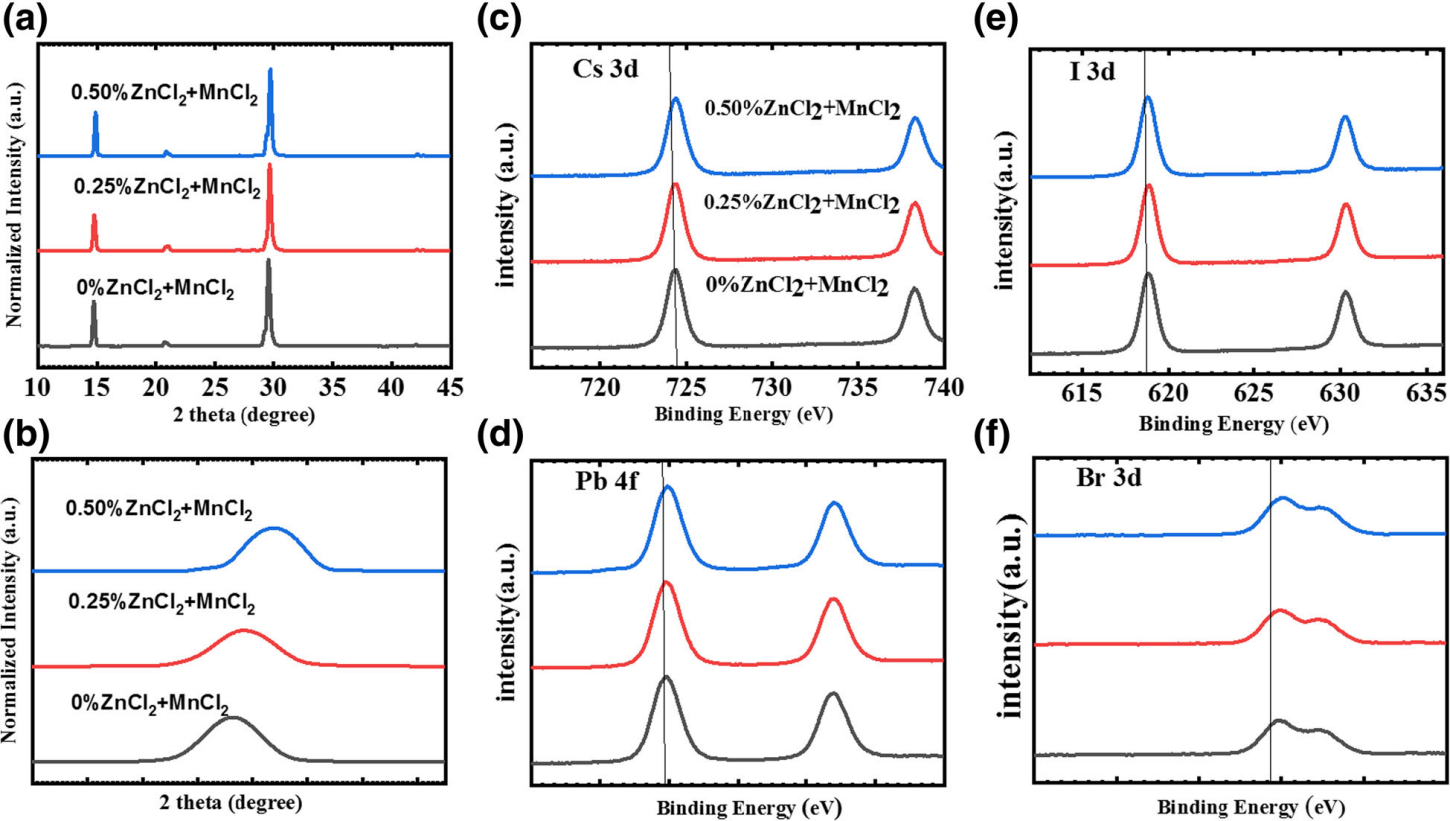
X-Ray diffraction (XRD) patterns (**a**) and the enlarged region of (100) peaks (**b**) for the CsPbI_2_Br-ZnCl_2_-MnCl_2_ films. XPS spectra of the CsPbI_2_Br-ZnCl_2_-MnCl_2_ films for Cs 3d (**c**), Pb 4f (**d**), I 3d (**e**), and Br 3d (**f**)

Figure 2a shows the XRD patterns of the CsPbI_2_Br films doped using different ZnCl_2_-MnCl_2_ concentrations. The thicknesses of all the CsPbI_2_Br films are controlled to be 350 nm. Figure 2b shows the enlarged region of the (100) peak. It can be seen that the peak of the CsPbI_2_Br-0.25% film shifts to a higher angle, indicating that the lattice constant is decreased. XPS analysis was performed to study the elemental composition and chemical state of the elements in the CsPbI_2_Br-ZnCl_2_-MnCl_2_ films. Figure 2c–f shows the XPS spectra of all components with the exception of ZnCl_2_ and MnCl_2_. As appeared in Fig. 2c, the Cs 3d range determines two peaks at 724.4 eV and 739.8 eV, which are apportioned to Cs 3d 3/2 and Cs 3d 5/2 of Cs+ cations, respectively. Figure 2d–f demonstrates that Pb 4f, I 3d, and Br 3d peaks shift to higher binding energy, which indicates that some Zn and Mn particles may replace certain Pb atoms located in B-sites of the perovskite, and therefore, the chemical bonding between halides and lead have been changed due to the ZnCl_2_-MnCl_2_ doping [35]. This is consistent with the above XRD analysis.

**Fig. 3:**
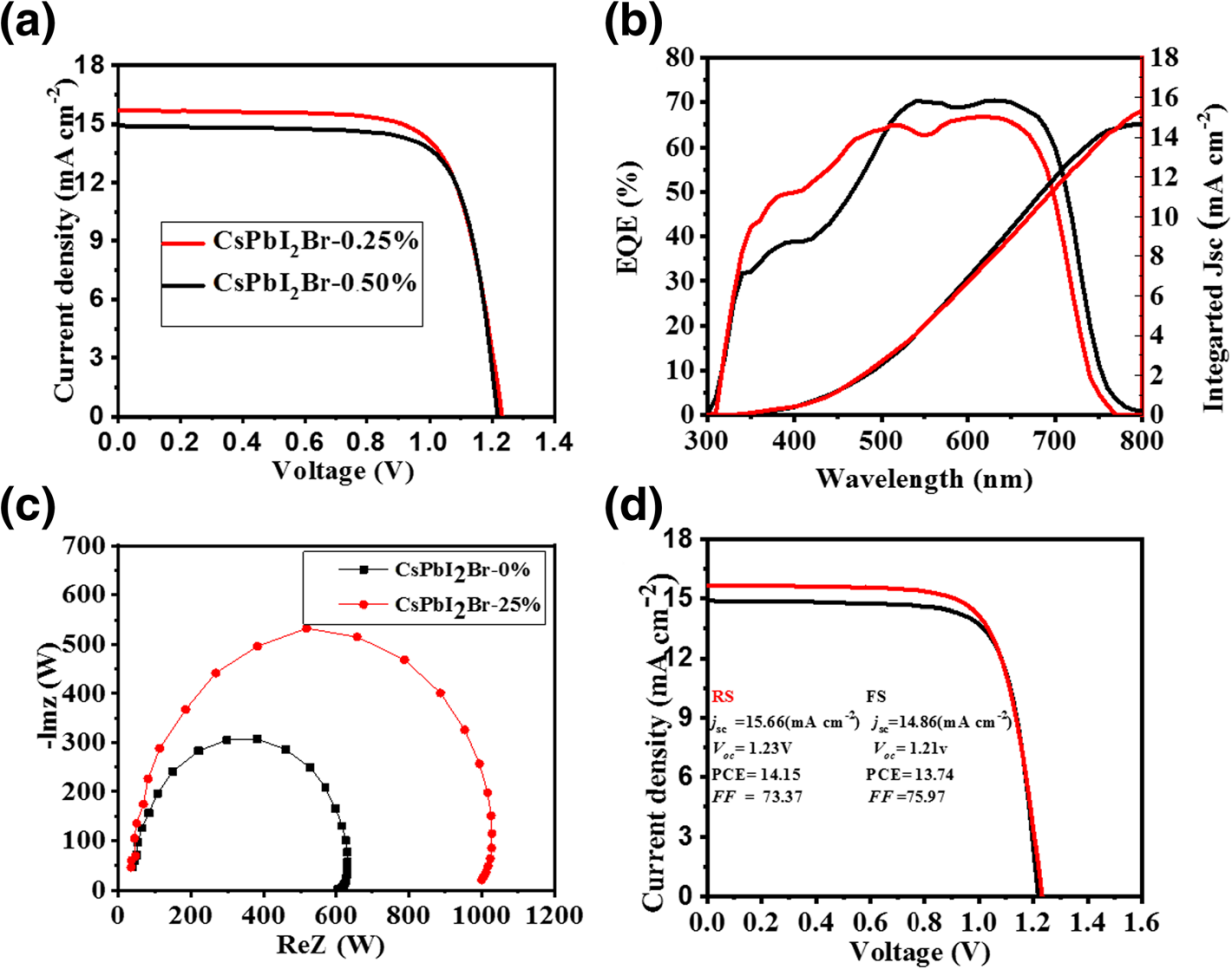
Lightweight *J–V* curves of the solar cells based on the CsPbI_2_Br-ZnCl_2_-MnCl_2_ films (**a**). EQE spectra and integrated *J*_sc_ of the solar cells based on the CsPbI_2_Br-0.25% (red) and the CsPbI_2_Br-0% (black) films (**b**). Nyquist plots (**c**). *J–V* characteristics under the directions of reverse and forward scanning (**d**)

The lightweight *J–V* curves of the cells based on the CsPbI_2_Br-ZnCl_2_-MnCl_2_ films are shown in Fig. 3a, and the relevant photovoltaic parameters are recorded in Table 1. The CsPbI_2_Br-0.25% device shows a champion PCE of 14.15%, with *J*_sc_ of 15.66 mA cm^−2^, *V*_oc_ of 1.23 eV, and FF of 73.37%, which are totally higher than those of the CsPbI_2_Br-0% device. We attribute this progress to enhanced film quality and reduced defects resulted from the ZnCl_2_-MnCl_2_ doping. External quantum efficiency (EQE) is done to verify the accuracy of *J*_sc_ completed from the *J–V* curve. As appeared in Fig. 3b, the EQE and interconnected *J*_sc_ of CsPbI_2_Br-0.25% device are greater than those of the CsPbI_2_Br-0% device. The interconnected *J*_sc_ of the CsPbI_2_Br-0.25% device is 15.66 mA cm^−2^, which is close to the *J*_sc_ of 14.86 mA cm^−2^ from the *J–V* bend. To research charge exchange properties of perovskite solar cells (PSCs), electrochemical impedance spectroscopy EIS spectra was completely abstracted as a function of voltage. The recombination resistance (*R*_rec_) was extracted from the diameter of the semicircle in the Nyquist plots. Figure 3c shows that the *R*_rec_ of the CsPbI_2_Br-0% and CsPbI_2_Br-0.25% devices are 620 Ω and 1016 Ω, respectively. The much larger *R*_rec_ for the CsPbI_2_Br-0.25% device originates from lower defect density, which indicates that charge recombination is effectively suppressed, leading to significantly improved *V*_oc_ and FF [37]. Figure 3d presents the typical *J–V* curves of the device with the best measured performance. Using the scanning directions of forward and reverse, the key parameters are summarized in the insert. It is noticeable that the device has very little hysteresis, as shown by the *J–V* curves.

**Table 1:** Comparison of the device parameters of the perovskite solar cells based on different CsPbI_2_Br-ZnCl_2_-MnCl_2_ films

	PCE [%]	*J*_sc_ [mA cm^−2^]	*V*_oc_ [*V*]	FF [%]
CsPbI_2_Br-0.25% RS	14.15	15.66	1.23	73.37
CsPbI_2_Br-0.25% FS	13.74	14.86	1.21	75.97
CsPbI_2_Br-0%	13.01	14.21	1.24	73.64
CsPbI_2_Br-0.50%	13.74	14.86	1.21	75.97
CsPbI_2_Br-0.125%	12.62	13.6	1.27	73.0

**Fig. 4:**
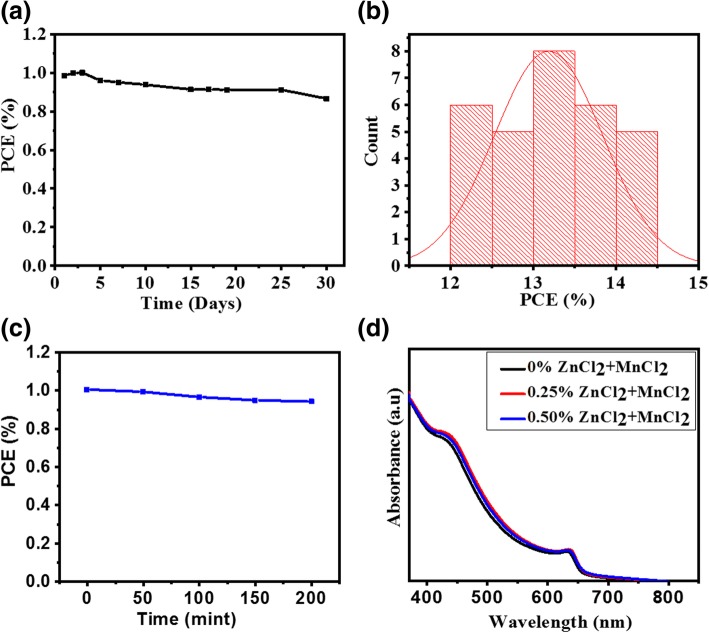
Normalized *V*_oc_, *J*_sc_, FF, and PCE for the solar cell based on the CsPbI_2_Br-0.25% film as a function of storage time (**a**). Histogram of power conversion efficiency values for 30 devices (**b**). Normalized PCE for the solar cell based on the CsPbI_2_Br-0.25% film as a function of thermal treatment time (**c**). Absorption spectra (**d**)

Finally, we studied the long-term stability of the perovskite solar cells PSCs based on the CsPbI_2_Br-0.25% film. The device was stored in a N_2_ glove box (20 °C in the dark). Figure 4a shows the normalized *J*_sc_, *V*_oc_, FF, and PCE as a function of the storage time. During the first 3 days, *J*_sc_, FF, and PCE all increase. This might be attributed to oxidation of spiro-OMeTAD by trace O_2_ (300–400 ppm) in the glove box. After 30 days, the PCE maintains 87% of its initial value and *V*_oc_ keeps almost constant. We expect that these results will help the development of cesium lead halide perovskites for next-generation photovoltaic. The histogram of 30 devices’ power conversion efficiency is shown in Fig. 4b, with statistics for photovoltaic parameters. Figure 4c shows the thermal stability of CsPbI_2_Br-0.25% device tested by heating the device at 80 °C for 150 min in the glove box, and after heating, the PCE of device maintains 96% of its initial value and *V*_oc_ keeps almost constant. The absorption spectra of ultraviolet–visible (UV–vis) were carried out to observe the photo physical characteristics for the CsPbI_2_Br-ZnCl_2_-MnCl_2_ films fabricated on glass substrate with a thickness of 70 nm. Figure 4d shows the absorption spectra of the CsPbI_2_Br-0.25% film. The absorption intensity is almost the same for all the CsPbI_2_Br-ZnCl_2_-MnCl_2_ films, and the absorption onset is around 600 nm. The above result suggests that the slight ZnCl_2_-MnCl_2_ doping hardly affects the band gap and the light absorption capacity of perovskite.

## Experimental Section

### Materials and Methods

#### Materials

The SnO_2_ were bought from Alfa Aesar. CsBr, ZnCl_2_, MnCl_2_, (DMSO), and (DMF) were bought from Sigma-Aldrich. spiro-OMeTAD and PbI_2_ were bought from Xi’an Polymer Light Technology Corp.

#### Device Fabrication

Initially, the ITO glasses were successively cleaned by applying detergent, isopropyl alcohol, acetone solvents about 20 min, and deionized water. The process is also followed by removing the substances remain in the substrates through oxygen plasma processing approximately for 10 min. The SnO_2_ were diluted in ultrapure water at a volume ratio of 1:6. Firstly, glass substrates were spin coated by SnO_2_ layer at 3000 rpm for 40 s, and then were annealed at 150 °C for 30 min. To prepare a perovskite precursor, CsBr, PbI_2_, ZnCl_2_, and MnCl_2_ were stoichiometrically dissolved in a mixed solvent of DMSO and DMF with a volume ratio of 1.4:1 to form a 1.0 M solution. The solution was filtered through a 0.22-μm pore PTFE filter, and then stirred at 70 °C for 2 h. The precursor solution was then spin coated on the SnO_2_/ITO substrate firstly at 1000 rpm with accelerating rate of 1000 rpm for 12 s, after that at 5000 rpm with accelerating rate of 3000 rpm not more than 30 s. Then, 100 μL of chlorobenzene (CB) were distilled onto the rotating substrate during the second step spin-coating with the time of 10 s before the end of the process. Afterwards, the film was first annealed at 50 °C for 1 min and then at 150 °C for 5 min. An HTL film was prepared by spin-coating spiro-OMeTAD solution onto the formed CsPbI_2_Br film at 4000 rpm with accelerating rate of 3000 rpm for 30 s. The spiro-OMeTAD solution consisted of 72.3 mg Spiro-OMeTAD, 17.5 μL bis (trifluoro methane) sulfonamide lithium salt (Li-TFSI) stock solution (520 mg Li-TFSI in 1 mL acetonitrile), 28.8 μL 4-tertbutylpyridine, and 1 mL chlorobenzene. At the end, the Au film with a thickness of 80 nm was deposited through thermal evaporation.

## Characterization

The Rigaku-2500 X-ray diffraction meter was used to measure the X-ray diffraction patterns. The top-view SEM images were attained using a scanning electron microscope (SEM, HITACH2100). Keithley 2420 was used to measure the solar cell *J–V* characteristics under AM 1.5 sunlight at an irradiance of 100 mW cm^−2^ provided by a solar simulator (Newport, Oriel Sol3A Class AAA, 94043A). The intensity of light was measured by monocrystalline silicon reference cell with a KG5 window (Newport, Oriel 91150). Impedance spectroscopy was measured by Zennium (Zahner). EQE was recorded using a Newport Oriel IQE-200 by a power source (Newport 300 W xenon lamp, 66920) with a monochromatic instrument (Newport Cornerstone 260). The device area is 0.044 cm^2^.

## Conclusions

In summary, we got inorganic CsPbI_2_Br solar cells by incorporating ZnCl_2_-MnCl_2_ into the CsPbI_2_Br precursor solution. When the ZnCl_2_-MnCl_2_ content achieves 0.25%, the device shows a champion PCE of 14.15%, with FF of 73.37%, *J*_sc_ of 15.66 mA cm^−2^, and *V*_oc_ of 1.23 eV. The enhanced photovoltaic performance is associated to improved surface morphology, reduced trap density, and suppressed charge recombination. This work could guide fundamental researches in the Cesium lead halide perovskites and promote their potential applications for solar cell.

### The Table of Contents Entry

A simple compositional engineering technique is used to improve the film quality and device performance. By incorporating MnCl_2_+ZnCl_2_ into the CsPbI_2_Br film, the CsPbI_2_Br perovskite solar cell attains an outstanding efficiency of 14.15% and good long-term stability. In addition, the fabrication process is highly reproducible and inexpensive.

## Abbreviations

DMF: N,N-dimethylformamide
DMSO: Dimethyl sulfoxide
EQE: External quantum efficiency
SEM: Scanning electron microscope
XPS: X-ray photoelectron spectroscopy
XRD: X-ray diffraction
